# DNA barcoding of the spider crab *Menaethius monoceros* (Latreille, 1825) from the Red Sea, Egypt

**DOI:** 10.1186/s43141-021-00141-2

**Published:** 2021-03-19

**Authors:** Mohamed Abdelnaser Amer

**Affiliations:** Zoology Department (Marine Biology Section), Faculty of Science, Al-Azhar University, Cairo, 11884 Egypt

**Keywords:** Epialtidae, Red Sea crabs, COI, Horny crab, 16S

## Abstract

**Background:**

Most spider crab species inhabiting the Red Sea have not been characterized genetically, in addition to the variation and complexity of morphological identification of some cryptic species. The present study was conducted to verify the identification of two morphotypes of the spider crab *Menaethius monoceros* (Latreille, 1825) in the family Epialtidae Macleay, 1838, collected from the Red Sea, Egypt. DNA barcoding of two mitochondrial markers, cytochrome oxidase subunit I (COI) and 16S, was used successfully to differentiate between these morphotypes.

**Results:**

DNA barcoding and genetic analyses combined with morphological identification showed that the two morphotypes were clustered together with low genetic distances ranged from 1.1 to 1.7% COI and from 0.0 to 0.06% 16S. Hence, this morphological variation is considered as individual variation within the same species.

**Conclusion:**

The present study successively revealed that genetic analyses are important to confirm the spider crab’s identification in case of morphological overlapping and accelerate the accurate identification of small-sized crab species. Also, DNA barcoding for spider crabs is important for better future evaluation and status records along the Red Sea coast.

## Background

The horny spider crab genus *Menathius* H. Milne Edwards, 1834 (Epialtidae Macleay, 1838) contains only two species: *Menaethius monoceros* (Latreille,1825) and *Menaethius oriantalis* (Sakai, 1969) [1–3]. They are common in the Indo-West Pacific extending from the Red Sea, Eastern Africa, and the Indian Ocean to Japan in the Pacific Ocean [1]. Recently, *M. monoceros* had its distribution extended and was recorded from the Mediterranean Sea by [4]. These species are known to inhabit weedy intertidal rocks in shallow waters [1, 2, 5, 6]. This genus can be distinguished by the following morphological characters: the presence of two small lobes or tubercles on the branchial margins, a small post-orbital lobe, propodi of the first walking leg is smooth ventrally, and with sexes similar in form. It is easy to differentiate *M*. *monoceros* from *M*. *oriantalis* by the presence of a slender rostrum, basally narrow; the dorsal branchial region has tubercles or rounded elevation; and walking legs are not carinate in *M*. *monoceros*. On the other hand, carinate walking legs are present in *M*. *orientalis* [1]. However, this genus has a geographical variation, as many authors have described several species which synonymized under *M. monoceros* [2] and listed by [7] treated in the list of Griffin and Tranter as synonyms [1].

Majidae is considered as a diverse group within brachyuran crabs, containing over 800 species, and was recently transferred to the superfamily Majoidea Samouelle, 1819 [3, 8–10]. Despite the revision of the majoid crabs by many authors [1–3, 11–14] throughout the Indo-West Pacific region and the list of [15] from the Arabian Gulf, some genera and species still have taxonomic confusion, and many workers have difficulty in identification using the keys within subfamilies to recognize the genera and species.

The eastern Egyptian coast of the Red Sea includes the eastern and western coasts of the Suez Gulf and the western Aqaba Gulf coast, with a total length of about 1300 km [16]. The Suez Gulf is considered as the boundary between Asia and Africa having an important role in the faunal migration between the Red Sea and the Mediterranean Sea [17] via the Suez Canal. The coasts of the Red Sea are represented by several habitats: soft, rocky, and coral reef habitats. These different habitats comprise suitable substrates as refuges and preferable habitats for different marine invertebrates.

The crabs of the Red Sea were listed and reviewed by [18–25]. Other later workers reviewed the majoids [11] and listed 32 species, as well as accounts of Red Sea majoids by [1, 26] who listed 46 spider crab species within Majoidea including 12 species in the family Epialtidae: *Acanthonyx elongatus* Miers, 1877; *A. dentatus* H. Milne Edwards, 1834; *Huenia heraldica* (De Haan, 1837) [= *Maja* (*Huenia*) *proteus* De Haan, 1839]; *Menaethiops contiguicornis* (Klunzinger, 1906); *M*. *dubius* Balss, 1929; *M. ninii* Guinot, 1962; *M*. *nodulosus* (Nobili, 1905); *Menaethius monoceros* (Latreille, 1825); *Perinia tumida* Dana, 1851; *Simocarcinus pyramidatus* (Heller, 1861); *S*. *simplex* (Dana, 1852); and *Xenocarcinus tuberculatus* White, 1847.

The recent revisions of brachyuran crabs depending only on morphological descriptions have many issues [27, 28]. And therefore, the use of DNA barcoding can help accelerate the identification of confused and even cryptic species [27, 29]. Molecular phylogenetics is a valuable tool to study the morphological evolution of decapod crustaceans, which may reflect their behaviors and distributions [27, 30–33].

Furthermore, using mitochondrial cytochrome oxidase subunit I gene in molecular studies in crustacean’s taxonomy is useful for species delimitation [29, 34]. Besides COI, the 16S rRNA gene is commonly used in constructing animal phylogeny because of the combination of the variable and conserved region with the same gene [30]. Also, the sequence lengths obtained with these two markers are in the range of sequences available in the GenBank database. With this in mind, the present study applied the DNA barcoding information of the spider crab *M. monoceros* exclusively from the Red Sea, Egypt, and successfully used phylogenetic analyses to confirm two possible morphotypes.

## Methods

### Sampling

A total of 20 specimens examined in the present study were collected from several locations across Red Sea Egyptian coasts: Hurghada (Marine Biology Station), Marsa Gabal El-Rosass, South Hammata, 9km north Marsa Alam, and south Bernis. Specimens were obtained attached to small weedy rocks in the shallow intertidal region.

The morphological identification followed [1, 35] and the key of Griffin and Tranter [2].

### DNA analyses

DNA was extracted from pieces of walking leg tissue for each crab specimen using a Qiagen DNA extraction kit (Germany); the final observed DNA concentrations with Nanodrop measuring machine were 19.2–27.7 ng μl. PCR amplification of 16S and COI markers was performed with 20-μl PCR cocktail volumes using 40 cycles (20μl) of 94°C for 40 s (denaturation), 48°C for 70 s (annealing), and 72°C for 90 s (extension), run on a PCR thermal cycler. PCR products were observed on 1.5 % agarose gels. Subsequently, purification of the formed single-band PCR products was done using a mixture of shrimp alkaline phosphate (SAP) and exonuclease (EXO1) protocol (37° C for 20 min followed by 83°C for 30 min.). Sequences were obtained by sending the cleaned PCR products to FASMAC (Yokohama, Japan). The acquired sequences were assembled using Bioedit and aligned and analyzed using the Mega 7 program [36]. The evolutionary history was inferred using the neighbor-joining method [37]. The evolutionary distances were computed for CO1 sequences using the Tamura 3-parameter method [38] while for 16S sequences using the Kimura 2-parameter method [39]. All accession numbers of the sequences used in genetic analyses are listed in Table 1. COI and 16S genes were amplified using universal primer sets following [40, 41], respectively.

**Table 1:** GeneBank accession number for the present examined materials of *M. monoceros* from the Red Sea, Egypt, and other available sequences from (http://www.ncbi.nlm.nih.gov) GenBank

Species	Reference code	Locality/distribution	GenBank accession #	
			CO1	16S
*M*. monoceros*	RCAZUE-Crus-Br.26151.13.1	Egypt/Red Sea	MW291632	MW291131
*M*. monoceros*	RCAZUE-Crus-Br.26151.13.2	Egypt/Red Sea	MW291633	MW291132
*M*. monoceros*	RCAZUE-Crus-Br.26151.13.3	Egypt/Red Sea	MW291634	MW291133
*M*. monoceros*	RCAZUE-Crus-Br.26151.13.4	Egypt/Red Sea	MW291635	MW291134
*M*. *monoceros*		Shimoda, Japan/Pacific		EU682804
*M*. *monoceros*		Shimoda, Japan/Pacific	EU682856	EU682805
*M*. *monoceros*		Christmas Island	GQ260899	
*M*. *orientalis*		French Polynesia/Pacific	GQ260900	

*Present study materials

## Results

### Systematic account

Epialtidae Macleay, 1838

*Menaethius* H. Milne Edwards, 1834

*Menaethius monoceros* (Latreille, 1825)

### Synonyms

*Pisa monoceros* Latreille, 1825:139-140.

*Menaethius monoceros*. Alcock, 1895: p. 197. -Klunzinger, 1906: p. 20. -Balss, 1924: p. 27. -Urita, 1926: p. 32. -Sakai, 1934: p. 294; 1936: p. 91, pl. 21, fig. 3; 1938: p. 263, pl. XXVI fig. 3 -Forest and Guinot, 1961:14, fig. 9a & b. -Sakai, 1965: 74-75, pi. 33 fig. 4. -Griffin, 1974:21.

### Materials examined

RCAZUE.Crus-Br.26151.13, four females, Hurghada, Marine Biology Station, Red Sea, Egypt, 27° 17′ 18.9″ N, 33° 45′ 46.6″ E, and other additional specimens previously collected during the period from April 1996 to February 1997 from the Red Sea Egyptian coasts are listed in Table 2.

**Table 2:** Morphometric measurements for the examined materials of *M. monoceros*, Red Sea, Egypt

Code	CL (mm)	CW (mm)	Abd.W (mm)	RL (mm)	RL/CL %	Sex	Analyses	Locality	Date of collection
RCAZUE-Crus-Br.26151.13	13.4	8.6		4	29.85	♀	Morphology and molecular	Hurghada, Marine Biology Station	February 2018
RCAZUE-Crus-Br.26151.13	14.1	9.3		4.1	29.08	♀			
RCAZUE-Crus-Br.26151.13	6.9	4		2.2	31.88	♀			
RCAZUE-Crus-Br.26151.13	5.6	3.2		1.4	25.00	♀			
RCAZUE-Crus-Br.26151.01	7.1	3.5	1.5	1.6	22.54	♀	Morphology	9km north Marsa Alam	19/4/1996
RCAZUE-Crus-Br.26151.01	7.1	4.6	2.7	1.9	26.76	♀			
RCAZUE-Crus-Br.26151.02	15.8	9.3	3.5	4.6	29.11	♂		Marsa Jabal Elrosas	20/4/1996
RCAZUE-Crus-Br.26151.02	12.8	8.1	2.6	3.7	28.91	♂			
RCAZUE-Crus-Br.26151.03	12	7.9	6.1	2.7	22.50	♀		67km north Marsa Alam	9/7/1996
RCAZUE-Crus-Br.26151.03	19.9	13.3	2.9	6.7	33.67	♂			
RCAZUE-Crus-Br.26151.03	14.6	11.1	7.7	3.7	25.34	♀			
RCAZUE-Crus-Br.26151.03	11.7	7.3	1.6	2.8	23.93	♂			
RCAZUE-Crus-Br.26151.04	13.5	9.5	6.1	3.4	25.19	♀		34km north Marsa Alam	10/7/1996
RCAZUE-Crus-Br.26151.04	11.1	6.9	2.7	3.2	28.83	♂			
RCAZUE-Crus-Br.26151.04	7	6.1	2.3	1.9	27.14	♀			
RCAZUE-Crus-Br.26151.04	7.7	4.5	1.3	2.3	29.87	♂			
RCAZUE-Crus-Br.26151.05	20.2	13.2	3.8	6.4	31.68	♂		18km north Marsa Alam	18/7/1996
RCAZUE-Crus-Br.26151.06	11.1	7	2.2	3.1	27.93	♂		3km south Bernis	19/2/1997
RCAZUE-Crus-Br.26151.07	16.8	11.2	3.6	4.6	27.38	♂		3km south Hamata	20/2/1997
RCAZUE-Crus-Br.26151.07	14.9	10.3	2.6	4	26.85	♂			
RCAZUE-Crus-Br.26151.07	14.8	10.2	7.1	4.2	28.38	♀			
RCAZUE-Crus-Br.26151.07	13.1	8.9	6.6	3.2	24.43	♀			
RCAZUE-Crus-Br.26151.07	12	8.3	5.9	2.5	20.83	♀			

### Remarks

**Fig. 1:**
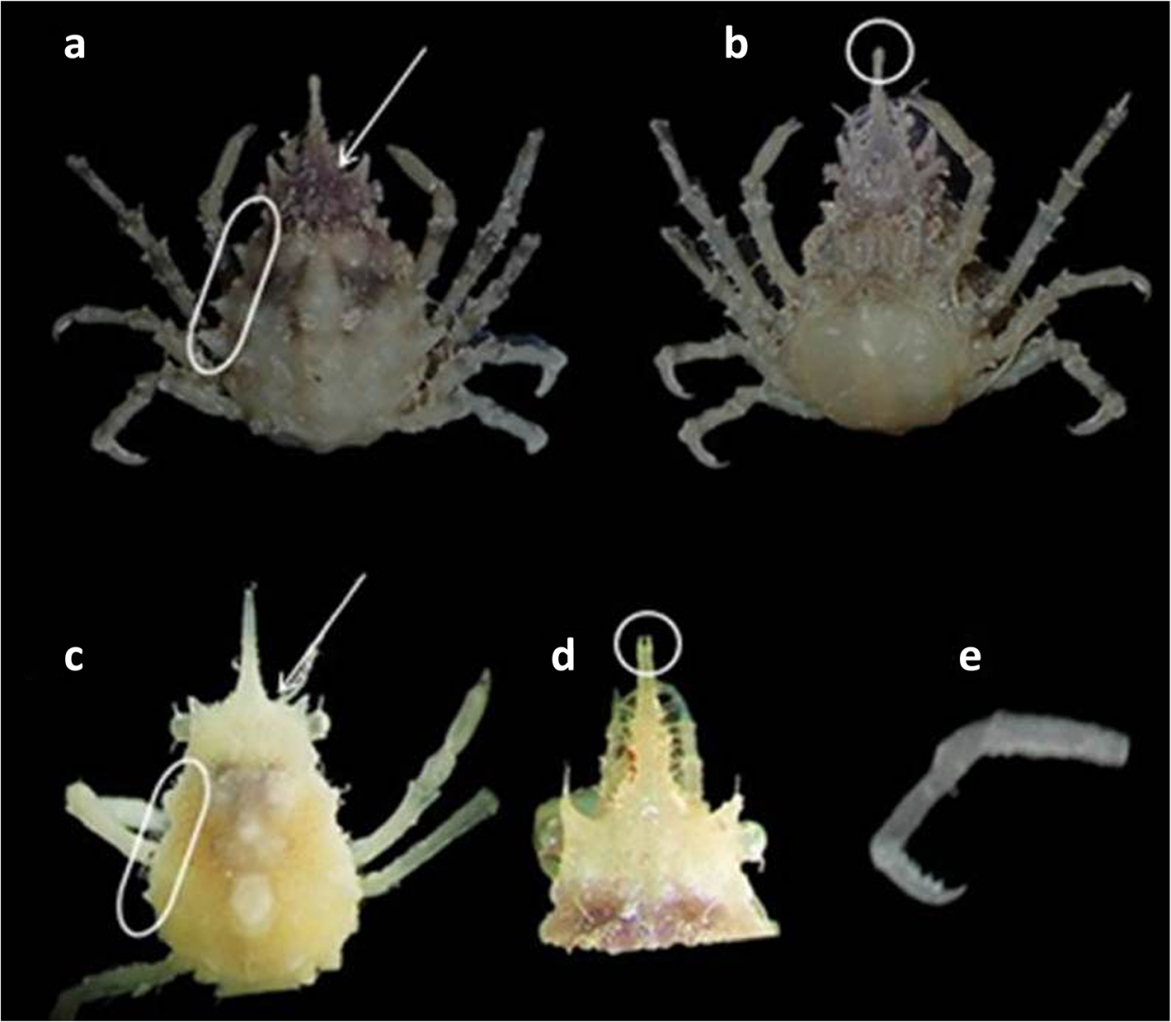
Two morphotypes of *M*. *monoceros*, 27° 17′ 18.9″ N, 33° 45′ 46.6″ E, Hurghada, Red Sea, Egypt. RCAZUE-Crus-Br.26151.13 (**a**, **b** Cl=13.4mm; **c**–**e** Cl= 6.9mm)

**Fig. 2:**
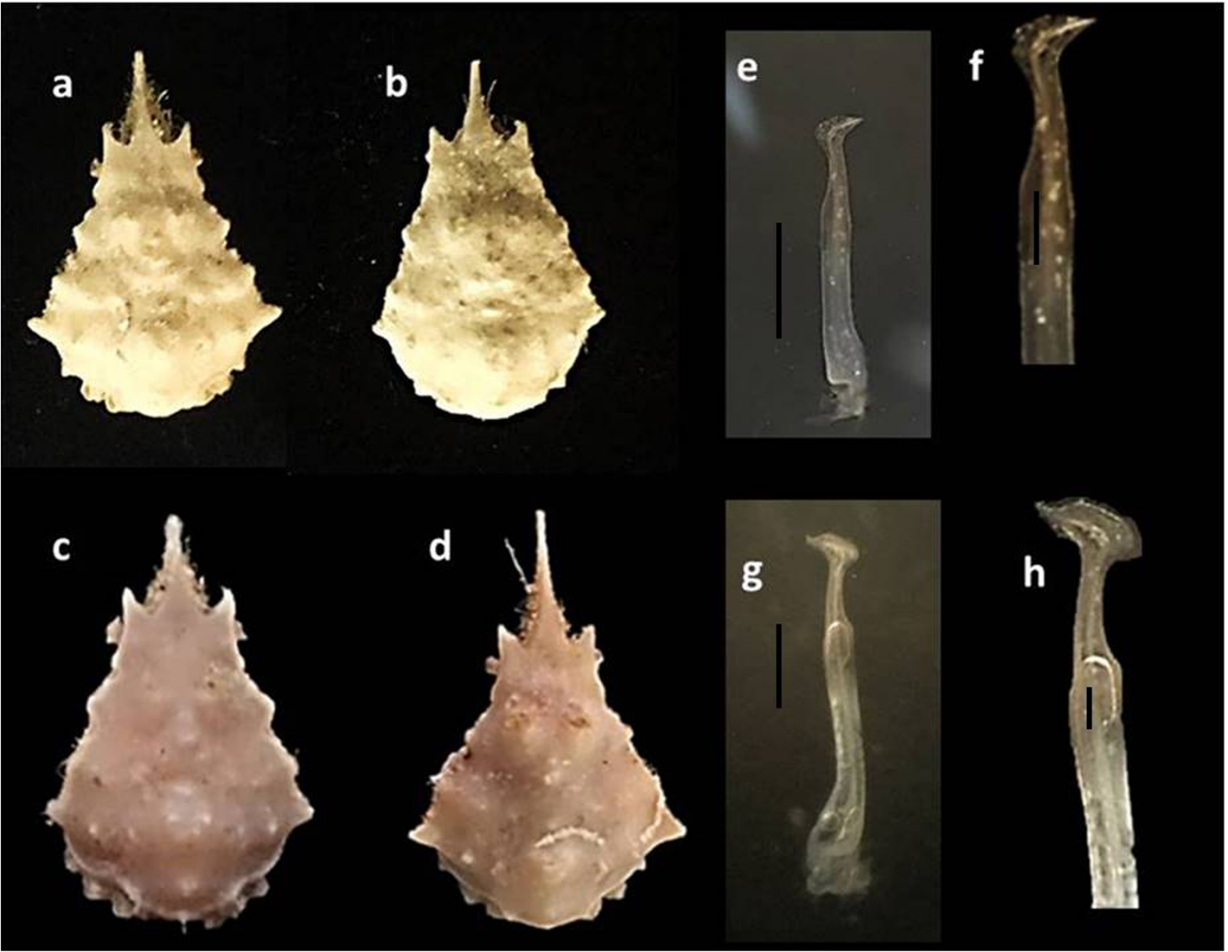
*M*. *monoceros*. **a**–**d** Dorsal surface of carapace. **e**–**h** First male pleopod. **a** RCAZUE-Crus-Br.26151.07. **b** RCAZUE-Crus-Br.26151.06. **c**, **e**, **f** RCAZUE-Crus-Br.26151.03. **d**, **g**, **h** RCAZUE-Crus-Br.26151.05. Scale bars=1mm

Despite the similarity between sexes in some characters for the genus *Menaethius* described by [2] and the agreement for some characters described by [35] especially in the rostrum (slender or some time bifurcated) which were similar to the present all specimens, there were two differences between the present specimens. We observed two morphotypes. The first morphotype consisted of two specimens (CL, 5.6 and 6.9) with slightly bifurcated rostrum in appearance and obtuse lobes or tubercles in the branchial margins versus the other morphotype (CL, 13.4 and 14.1) which had a rounded tip of the rostrum and obvious lobes or tubercles on the branchial margins. On the other hand, the comparison of all descriptions of synonymized names for *M*. *monoceros* includes a variety of morphological differences. Moreover, there is a variation in the morphology of this species across its geographical distribution [2], in addition to slight differences observed between two populations examined by [6] from two localities at the northern Red Sea. In addition, there has been a variation reported in carapace tuberculation in different sites (Figs. 1 and 2). All of these previous reasons make it difficult to accurately identify the morphotype of the present specimens, making DNA analyses important to reach a precise identification.

### Molecular results

**Fig. 3:**
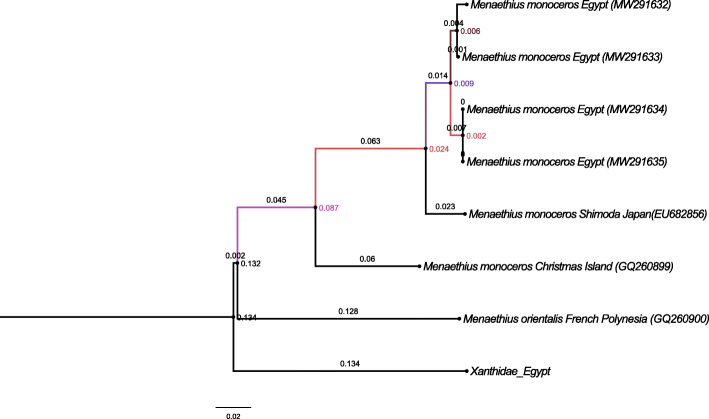
Evolutionary relationships of selected taxa sequences with COI using the Neighbor-Joining method with a bootstrap test (1000 replicates). Evolutionary distances were computed using the Tamura 3-parameter method; the rate variation among sites was modeled with a gamma distribution (shape parameter = 1)

**Fig. 4:**
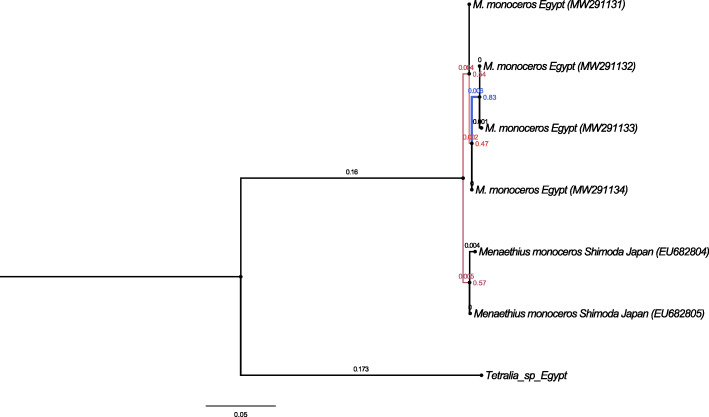
Evolutionary relationships of taxa sequences with 16S marker using the Neighbor-Joining method with a bootstrap test (1000 replicates). Evolutionary distances were computed using the Kimura 2-parameter method; the rate variation among sites was modeled with a gamma distribution (shape parameter = 1)

The genetic analyses of the two markers used, 16S and COI (Figs. 3 and 4 and Tables 3 and 4), revealed that the two closely related morphotypes of *M*. *monoceros* showed slight genetic differences. The COI neighbor-joining tree separated the two morphotypes, but the genetic differences were only 1.1 to 1.7% (Table 3). As shown in Table 4, the genetic distance for 16S sequences between the two morphotypes was negligible, ranging from 0.0 to 0.6%. These results indicate that the genetic differences are not enough to separate these morphotypes into different species based on a cutoff OTU threshold of 97% [42], and thus, I consider this variation as individual variation within the same species.

**Table 3:** Pairwise genetic distances between the studied specimens’ sequences compared with the sequences obtained from GenBank COI sequences of *M*. *monoceros*

Number	Distances	1	2	3	4	5	6	7
1	*M. monoceros* Egypt (MW291632)							
2	*M. monoceros* Egypt (MW291633)	0.006						
3	*M. monoceros* Egypt (MW291634)	0.017	0.011					
4	*M. monoceros* Egypt (MW291635)	0.017	0.011	0.000				
5	*M. monoceros* Christmas Island (GQ260899)	0.145	0.146	0.146	0.146			
6	*M. monoceros* Shimoda Japan (EU682856)	0.048	0.041	0.043	0.043	0.142		
7	*M. orientalis* French Polynesia (GQ260900)	0.255	0.260	0.260	0.260	0.232	0.252	
8	Xanthidae Egypt	0.260	0.260	0.264	0.264	0.241	0.282	0.264

**Table 4:** Pairwise genetic distances between the studied specimens’ sequences compared with the sequences obtained from GenBank 16S sequences of *M*. *monoceros*

Number	Distances	1	2	3	4	5	6	7
1	*M. monoceros* Egypt (MW291131)							
2	*M. monoceros* Egypt (MW291132)	0.006						
3	*M. monoceros* Egypt (MW291133)	0.006	0.000					
4	*M. Monoceros* Egypt (MW291134)	0.000	0.006	0.006				
5	*M. monoceros* Shimoda Japan (EU682804)	0.014	0.021	0.021	0.014			
6	*M. monoceros* Shimoda Japan (EU682805)	0.014	0.020	0.020	0.014	0.006		
7	*Tetralia* sp. Egypt	0.389	0.384	0.384	0.389	0.422	0.405	

### Habitat

The present specimens were observed and obtained from weedy shallow intertidal small rocks at depths of 0–2m.

### Life coloration

Larger specimens have a gray color on the legs and branchial regions with light orange color on the hepatic and cardiac prominence, but the smaller specimens are white to creamy all over the carapace and legs.

### Distribution

Red Sea, throughout the Indo-West Pacific from South Africa to Japan, Australia, and Tahiti, recently recorded from the Mediterranean Sea.

## Discussion

Two or more species imprecisely classified or distinguished morphologically under one species name usually called cryptic species [43]. Moreover, the great diversity among the Indo-West Pacific fauna of majoid crabs was treated morphologically by [1, 2]. Despite the provided key by Grifin and Tranter, many problems still face many workers in the identification of majoid crabs, and as such, this confusion has caught the attention of many workers [12–14].

The genus *Menaethius* has a variation in characters described by [35] in between its only two species (*M*. *monoceros* and *M*. *oriantalis*). The dorsal surface of carapace is mounted with a variable number of tubercles on the gastric and cardiac regions, the male pseudorostrum is usually relatively longer than females, and the tip is somewhat rounded and sometimes bifurcated. The branchial margins often have two teeth and sometimes with numerous tubercles. Some variation of the previous characters was also observed in the present studied specimens (Figs. 1 and 2) which leads to doubtful morphological identification and needs confirmation with genetic analyses.

It is known that delimitation of single species for discovering new species is a modern topic of discussion in systematics [44]. Thus, DNA barcoding for animal species is considered as a somewhat new and important taxonomic tool [45]. Moreover, DNA barcoding can speed the morphological identification of described species [28]. The present study investigated four specimens resembling the original description with variation in some characters and appearance of two morphotypes. Molecular analyses showed that the genetic distance difference between them ranged from 0.6 to 1.7% (Tables 3 and 4), which is low and not enough to separate them into different species. However, the genetic differences in the distance between the present studied specimen’s sequences from those from Shimoda, Japan (EU682804, EU682856), obtained from GenBank ranged between 1.4 and 2.1%, for 16S marker and between 1.42 and 4.8% in COI. These larger differences may be attributed to the geographical variation between both populations (Red Sea versus Pacific). Finally, DNA barcoding proved to increase the identification speed of small crab species, and thus, we consider it has a potential to be an important tool combined with morphology in taxonomic studies.

## Conclusions

Most small-sized crab species inhabiting the Red Sea and associated with seaweeds are difficult to identify morphologically, and thus, the present study used two DNA markers to confirm the identity of two morphotypes of the spider crab *Menaethius monoceros*. DNA barcoding and phylogeny revealed that there was a variation within the same species. Also, the present study provides baseline DNA data for one species inhabiting the Red Sea, which has recently been reported as migrated to the Mediterranean Sea. Our data will be important in further future investigations.

## Data Availability

The datasets used and analyzed during the current study are presented in the article and additional genetic data (sequences) available and deposited in the GenBank with an accession number.

## Abbreviations

Cl: Carapace length
CW: Carapace width
RL: Rostrum length
Abd.W: Abdominal width
COI: Cytochrome oxidase subunit I
16S: Ribosomal subunit rRNA
